# Arthroscopic Remplissage Before Bankart Repair With All-Suture Anchor Mattress Fixation in the Beach-Chair Position

**DOI:** 10.1016/j.eats.2024.103206

**Published:** 2024-08-22

**Authors:** Alexander R. Markes, Luke Sang, Elliott Cole, Brian T. Feeley

**Affiliations:** Department of Orthopaedic Surgery, University of California-San Francisco, San Francisco, California, U.S.A.

## Abstract

A common procedure for treatment of Hill-Sachs lesions in the setting of anterior shoulder instability is arthroscopic remplissage. Remplissage consists of using the posterior capsule and infraspinatus tendon to fill the Hill-Sachs lesion and convert it into an extra-articular defect. Previous versions of this technique have not specified the timing in which remplissage and Bankart repair occur and have been performed with the patient in the lateral decubitus position. In this Technical Note, we describe our technique where we perform the remplissage before Bankart repair using all-suture anchor mattress fixation with the patient in the beach-chair position. By performing the remplissage before Bankart repair, the shoulder is reduced to allow for easier execution of the remplissage and reduce difficulties that might prevent its completion if done after Bankart repair. Further, by completing remplissage before Bankart repair in the beach-chair position, the humeral head is moved posteriorly with the cuff to allow for better access for the following labral repair and allows for the standardization between arthroscopic and open shoulder instability management.

The most common approach to the management of a Hill-Sachs lesion (HSL) in the setting of anterior shoulder instability is through an arthroscopic remplissage.^1^ The remplissage technique involves using the posterior capsule and infraspinatus tendon to fill the HSL and turn it into an extra-articular defect. This procedure has been modified by multiple groups and has overall led to good functional outcomes.^2^^,^^3^

This Technical Note describes a Bankart repair with remplissage technique that is modified from recent revisions of this procedure. We complete arthroscopic remplissage before Bankart repair using all-suture anchor mattress fixation with the patient in the beach-chair position (Video 1). The timing of our technique creates the advantage of reducing the shoulder to allow for an easier remplissage and reduce difficulties that might prevent the completion of remplissage if done after Bankart repair. Previous remplissage techniques also have been largely described with the patient in the lateral decubitus position, given the concern for access to the anterior labrum in the beach-chair position. However, by completing the remplissage, we demonstrate that the humeral head moves posteriorly with the cuff to allow for better access for subsequent labral repair. Furthermore, performing arthroscopic stabilization with the patient in the beach-chair position allows for standardization of setup across open and arthroscopic procedures.

## Surgical Technique

**Fig 1:**
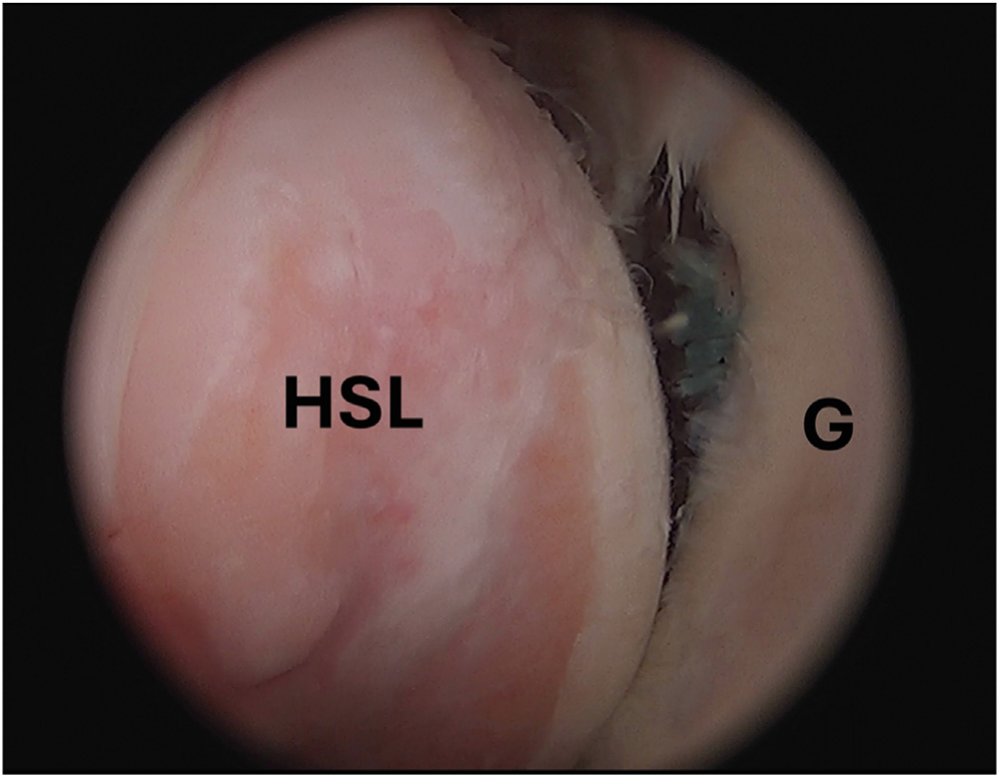
Arthroscopic image of the left shoulder viewed from a posterior portal with the patient in the beach-chair position demonstrating an HSL. (G, glenoid; HSL, Hill-Sachs lesion.)

A standard posterior portal is created, and diagnostic arthroscopy is performed to confirm lack of glenoid bone loss, quality of anterior inferior labrum, and size of the HSL (Fig 1). The anterior portal is made high in the rotator interval for ease of future capsulolabral mobilization. In cases of anterior shoulder instability, the humerus will be subluxed anterior relative to the glenoid with the HSL easily visualized from the posterior portal with the arm forward flexed to 20° and slight traction placed via the hydraulic arm holder. It is our protocol to commonly complete the remplissage before Bankart repair, given the good visualization of the lesion while the head is displaced anteriorly.

**Fig 2:**
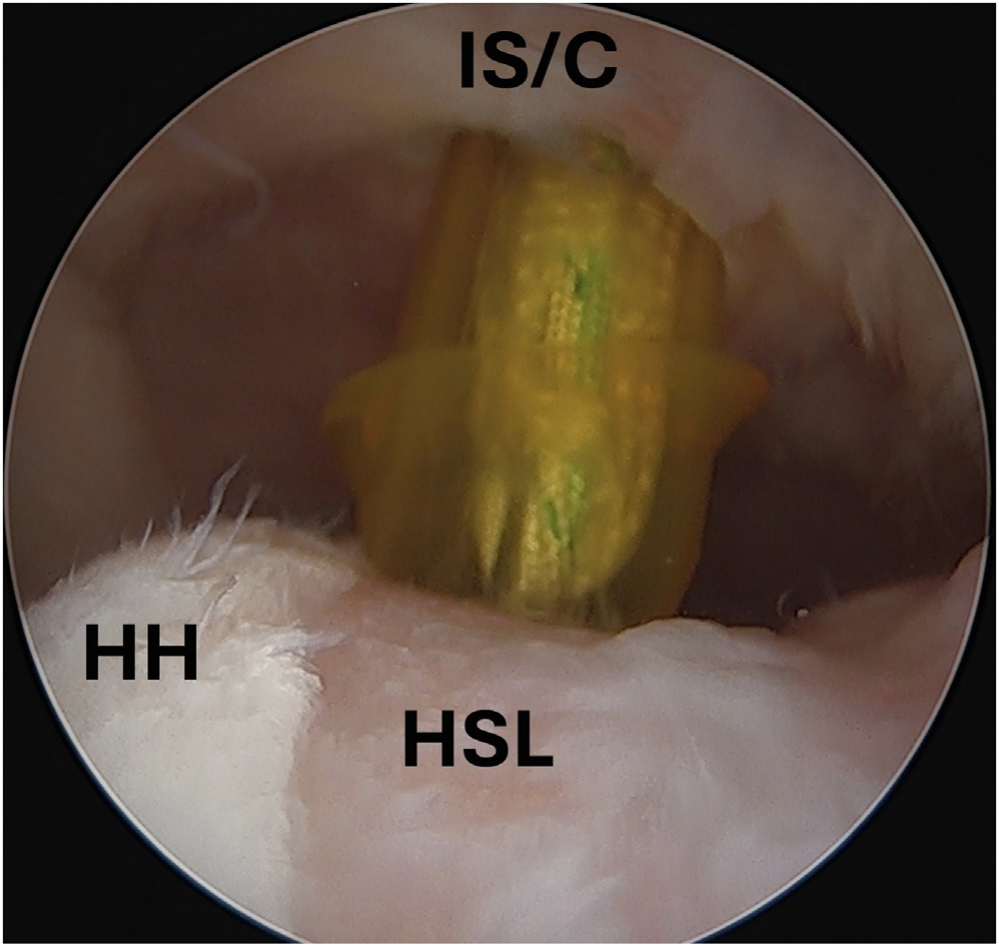
Arthroscopic image of the left shoulder viewed from an anterior portal with the patient in the beach-chair position demonstrating placement of a self-punching 2.8-mm double-loaded all-suture anchor (Y-knot RC; Conmed Corp, Utica, NY) into the HSL. (HH, humeral head; HSL, Hill-Sachs lesion; IS/C, infraspinatus/capsule.)

**Fig 3:**
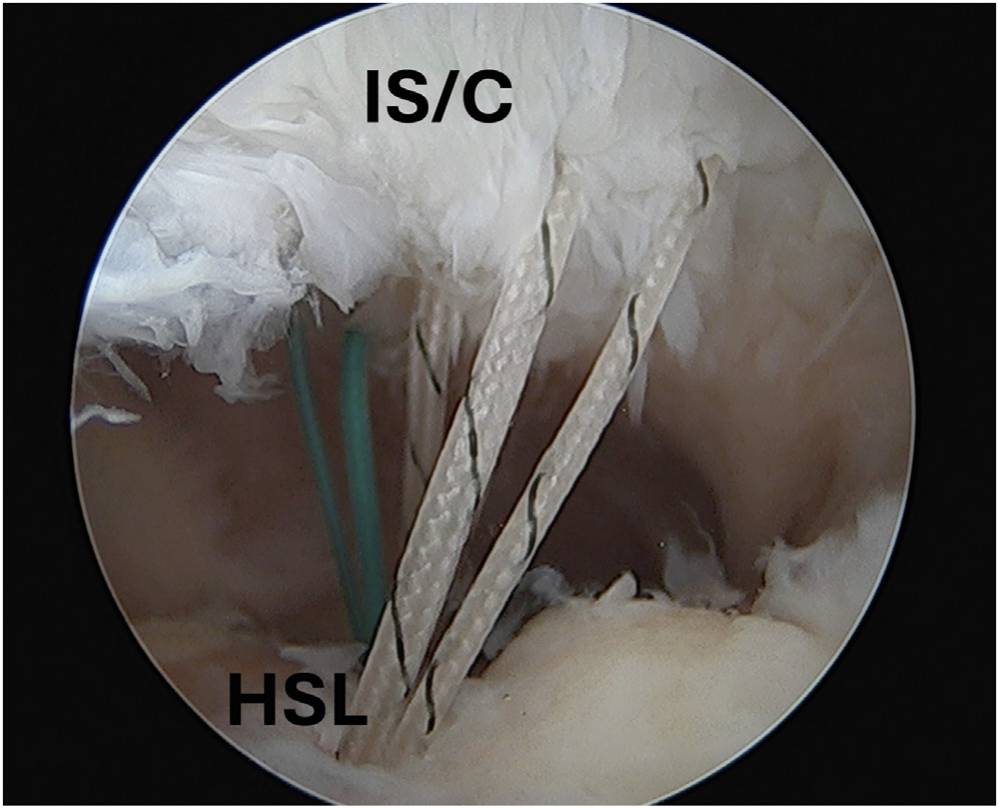
Arthroscopic image of the left shoulder viewed from an anterior portal with the patient in beach-chair position demonstrating 2 all-suture anchors placed at the anterior and posterior extent of the HSL with sutures passed through the infraspinatus and capsule. (HSL, Hill-Sachs lesion; IS/C, infraspinatus/capsule.)

**Fig 4:**
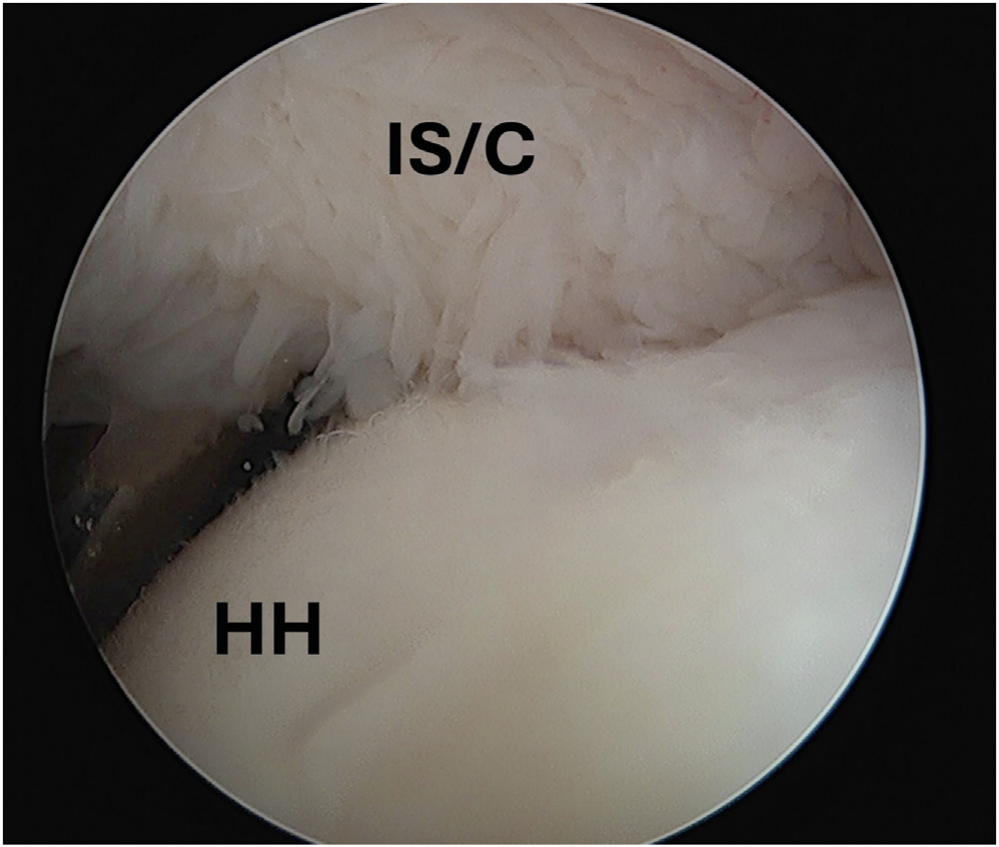
Arthroscopic image of the left shoulder viewed from an anterior portal with the patient in the beach-chair position demonstrating completed remplissage with infraspinatus capsulotenodesis filling the entire Hill-Sachs lesion. (HH, humeral head; IS/C, infraspinatus/capsule.)

Viewing the HSL from the anterior portal, the posterior portal is used as a working portal with a 5.0-mm cannula. A No. 15 blade is used to expand the posterior portal skin incision 1 cm, which is then carried down to the subdeltoid space without violating the underlying infraspinatus tendon. The space is expanded with use of a hemostat for later ease of passing and tying sutures. The lesion is prepared for optimal healing potential through use of rasp. Using the cannula of posterior portal, a self-punching 2.8-mm double-loaded all-suture anchor (Y-knot RC; Conmed Corp, Utica, NY) and a self-punching 2.8-mm triple-loaded all-suture anchor (Y-knot RC; Conmed Corp) are respectively placed at the anterior and posterior extent of the HSL (Fig 2). A sharp tip grasper (BirdBeak; Arthrex, Naples, FL) is used penetrate through the infraspinatus and capsule through the same incision as the posterior portal just next to the cannula and grab sutures from the anterior most anchor. This process is repeated with both sutures from the posterior most anchor. In this case, using three mattress sutures to fully cover the footprint of our HSL would acceptable. The unused sutures are unloaded, leaving 3 sutures passing through the infraspinatus and capsule (Fig 3). These sutures are then successively tied down in a mattress fashion with excess suture cut, completing the remplissage (Fig 4).

**Fig 5:**
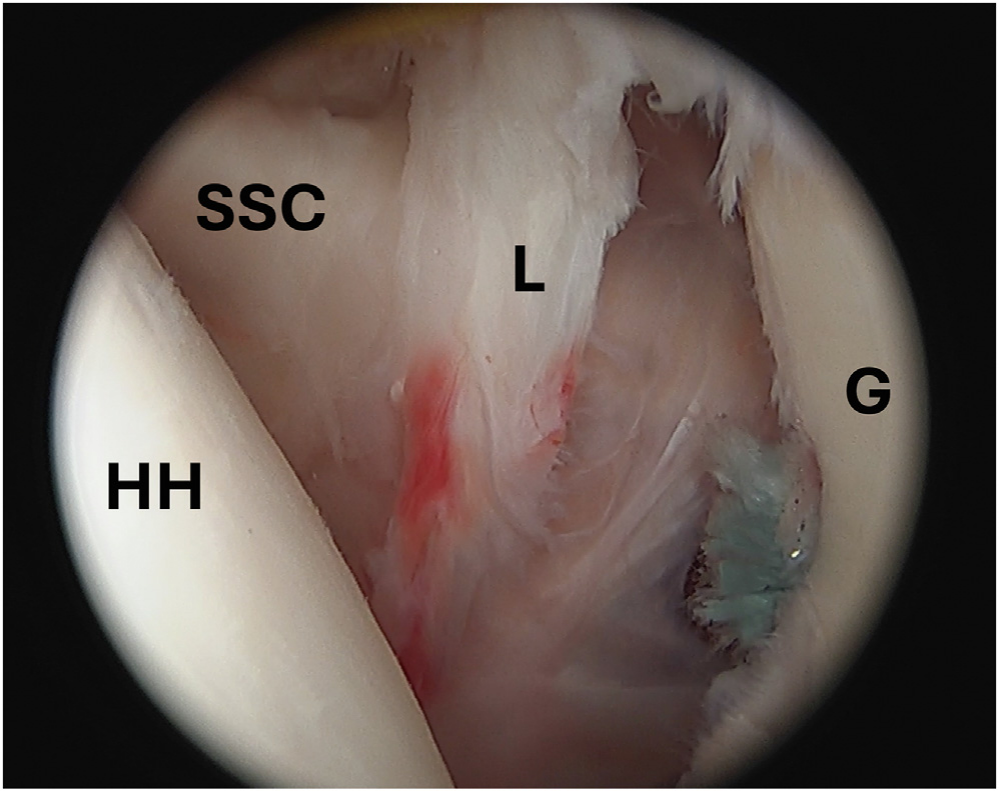
Arthroscopic image of the left shoulder viewed from a posterior portal with the patient in the beach-chair position demonstrating adequate visualization of anterior inferior labral tear from the glenoid rim after completion of previous remplissage. (G, glenoid; HH, humeral head; L, labrum; SSC, subscapularis tendon.)

**Fig 6:**
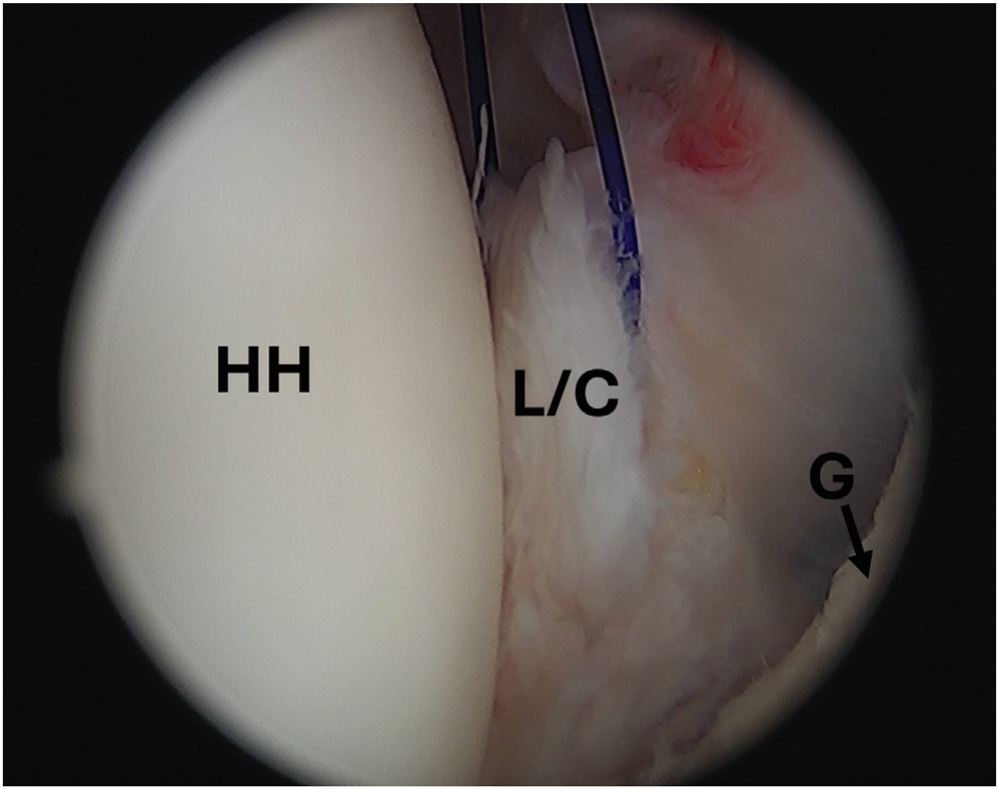
Arthroscopic image of the left shoulder viewed from a posterior portal with the patient in the beach-chair position demonstrating traction suture being used to allow for ease of access to anterior labrum and capsule for the capsulorrhaphy. (G, glenoid; HH, humeral head; L/C, labrum/capsule.)

With the remplissage complete, the camera is returned to the posterior portal, and the humerus will be reduced posteriorly, improving exposure to the anterior inferior labrum for capsulorrhaphy (Fig 5). Using the anterior portal, the capsulolabral complex is mobilized using a sharp periosteal elevator. An accessory anteroinferior portal is created under direct visualization lateral in the rotator interval just superior to the subscapularis tendon. A grasper is used from the anterior portal to lift the capsulolabral complex, allowing easier passage of the curved suture passer from the anteroinferior portal through anteroinferior capsule and labrum. A nitinol wire used to shuttle a traction suture through the capsulolabral complex (Fig 6). The curved suture passer is then used again to shuttle a nitinol wire through the capsulolabral complex at the 7-o’clock position, which is grabbed and pulled out the anterosuperior canula to be later used to shuttle repair sutures through the capsulolabral complex.

**Fig 7:**
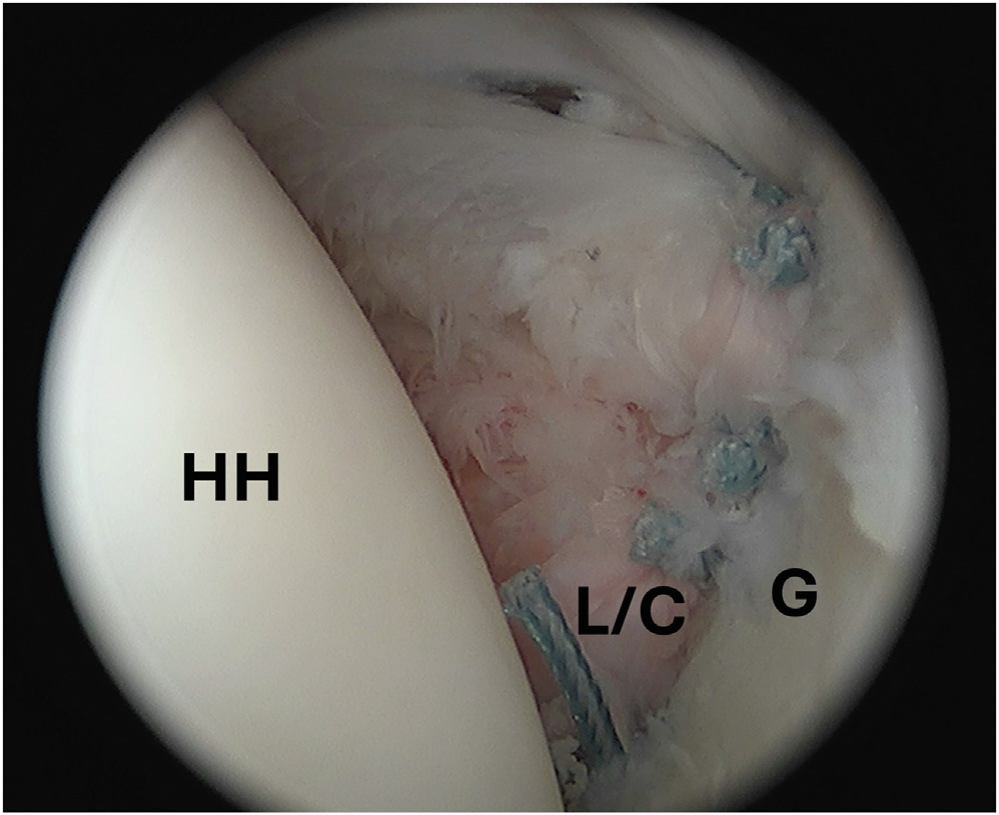
Arthroscopic image of the left shoulder viewed from a posterior portal with the patient in beach chair position demonstrating completed Bankart repair with 4 knotless, all-suture anchors in a simple stitch configuration (1.8-mm FiberTak; Arthrex). (G, glenoid; HH, humeral head; L/C, labrum/capsule.)

The number of anchors and amount of capsular shift should be determined on the basis of a variety of factors, such as the degree of capsular laxity, the severity of instability and labral injury, and the extent of the tissue mobilization. We commonly repair the capsulolabral sleeve with at least 3 knotless, all-suture anchors in a simple stitch configuration (1.8-mm FiberTak; Arthrex) as previously described.^4^ In this case, we decided to place 4 anchors to complete the repair (Fig 7).

### Postoperative Protocol

After surgery, the patient is placed in a shoulder immobilizer with abduction pillow for 6 weeks. From 0 to 2 weeks, Codman’s pendulum exercises are allowed. From 2 to 6 weeks, forward elevation to 120° is allowed. At week 6, the sling is discontinued, and active motion is initiated with progression to 85% of full range of motion by 12 weeks, at which time strengthening is begun. Patients are cleared for noncontact sports at 4 months and contact sports at 6 months depending on progression with sports-specific training.

## Discussion

This Technical Note comprehensively describes a technique in which remplissage occurs before Bankart repair in the beach-chair position, differing and building on recent modifications to this procedure.^2^ The order in which Bankart repair and remplissage occurs has not been strictly defined. Other techniques have already suggested the importance of at least placing HSL anchors for later remplissage to avoid anterior forces on the humeral head that might disrupt completed Bankart repair.^2^ This Technical Note describes a technique in which complete remplissage occurs before Bankart repair with the advantage of positioning the humeral head more posteriorly with the rotator cuff to allow for better access and visualization during subsequent labrum repair. This is particularly important when addressing anterior shoulder instability in the beach-chair position to have easier access to the anteroinferior glenoid and labrum. Further, the timing of this technique reduces the shoulder to allow for easier completion of the entire remplissage and likely increase rates of remplissage completion compared with doing so after Bankart repair. If the Bankart repair is performed first, additional complications such as Bankart repair disruption, anchor failure, impaired reduction, and improper suture management could all increase the difficulty of the following remplissage and thus impair its completion.^5^ Studies have shown a significantly greater risk for recurrent instability for patients who do not receive remplissage in conjunction to Bankart repair compared with those who do, making remplissage completion crucial.^3^^,^^6^, ^7^, ^8^, ^9^

In addition, although previous articles detailing remplissage have mostly been performed in the lateral decubitus position, this technique demonstrates a repair in the beach-chair position.^2^^,^^10^ A recent study by Yow et al.^11^ showed that there were no differences in rates of instability recurrence or revision surgeries for anterior shoulder stability arthroscopic procedures between the lateral decubitus or beach-chair positions. Meanwhile, the beach-chair position offers additional benefits of an easier setup and ability to convert to an open procedure (Table 1).^11^ This standardization of the beach-chair setup for both open and arthroscopic management of shoulder instability can limit confusion across members of the surgical team and possibly increase efficiency throughout various procedures.

**Table 1:** Pearls and Pitfalls

Pearls	Pitfalls
Expanding subdeltoid space around cannula with hemostat allows for single posterior incision for anchor placement and suture passing during remplissage Arm forward flexed with slight traction in a hydraulic arm holder allows anterior humeral head subluxation for easier access to the posterior humerus Traction sutures allow for easier access to anteroinferior labrum tissue during capsulorrhaphy	Ensure sutures are tied down in the subdeltoid space during remplissage Ensure spacing of anchors in HSL is appropriate to cover entire footprint with remplissage infraspinatus capsulotenodesis Directly visualize tensioning of capsulolabral complex to knotless all-suture anchor to ensure unintended loops in suture do not develop Ensure first pass of curved suture passer through capsule and labrum is adequately low to advance significant capsule and labrum onto edge of glenoid

HSL, Hill-Sachs lesion.

It is important to note that there are limitations of arthroscopic Bankart repair with remplissage (Table 2). In patients with greater than 10% to 15% of mean glenoid bone loss, arthroscopic soft-tissue stabilization with remplissage has been shown to have high rates recurring instability.^6^ In these cases, one must consider bony augmentation of the glenoid. However, even with bony augmentation of the glenoid, Calvo et al.^12^ demonstrated that patients with persistent off-track lesions after Latarjet for anterior shoulder instability were associated with a greater failure rate. Thus, one must still consider the use of remplissage as an adjunct for a persistently off-track HSL regardless of bony augmentation. In addition, return to sport in throwing or overhead athletes and loss of range of motion during capsulotenodesis with multiple remplissage anchors may be a concern with addition of remplissage to Bankart repair.^1^^,^^13^^,^^14^

**Table 2:** Advantages and Risks/Limitations

Advantages	Risks/Limitations
Initial anterior subluxation allows for easier posterior humerus visualization for completion of remplissage	Greater rates of recurrent instability in the setting of >10%-15% of glenoid bone loss
Completing remplissage repositions the humeral head more posteriorly, allowing for better anteroinferior access and visualization during labral repair	Surgeon and operating staff familiarity of performing arthroscopy in the beach-chair position
Avoids complications that increase difficulty and impair remplissage completion such as Bankart repair disruption, anchor failure and impaired reduction.	Potential decreased short-term range of motion and lower return to sport rates in throwing and overhead athletes with addition of remplissage to Bankart repair
The beach-chair position allows for easy standardization of shoulder instability management setup and limits confusion across members of the surgical team	

## Disclosures

The authors declare the following financial interests/personal relationships which may be considered as potential competing interests: B.T.F. reports Journal Editor for the *Journal of Shoulder and Elbow Surgery* and *Current Reviews in Musculoskeletal Medicine*. All other authors (A.R.M., L.S., E.C., B.T.F.) declare that they have no known competing financial interests or personal relationships that could have appeared to influence the work reported in this paper.

## Supplementary Data

Video 1 Outlined are step-by-step instructions to perform arthroscopic remplissage with all-suture anchor mattress fixation and an anterior labral repair with all-suture anchor knotless fixation. This procedure is performed on the left shoulder of a skeletally mature patient in the beach-chair position. Diagnostic arthroscopy is first performed though a posterior portal, confirming lack of glenoid bone loss, quality of anterior inferior labrum and labral tear, as well as to inspect the relative size and position of the Hill Sachs lesion (HSL). Next, viewing from the anterior portal and working through a posterior portal perpendicular to the HSL, all-suture anchors are placed at the terminal ends of the HSL. The posterior portal is enlarged to allow passing of sutures through separate intervals of capsulotendinous layer of infraspinatus, which are then tied, completing the capsulotenodesis. Switching the camera back to the posterior portal, the anterior portal is used to prepare the glenolabral junction. An anteroinferior portal is created through which a suture passer is used to pass a traction suture through the capsulolabral complex. A nitinol wire is shuttled through the capsulolabral complex at the 7-o’clock position. A knotless all-suture anchor is placed along the glenoid rim and the nitinol wire is used to shuttle the repair suture through capsulolabral junction. The repair suture is then shuttled through self-locking mechanism of the anchor, advancing the capsulolabral complex onto the glenoid to desired loop tension after removing the traction suture. This process is repeated 3 times completing the labral repair.
